# Progress in Dental Mechanism. No. 4

**Published:** 1868-02

**Authors:** 


### AMERICAN JOURNAL

OF

### DENTAL SCIENCE.

Vol. I. THIRD SERIES--FEBRUARY, 1868. No. 10.

### ORIGINAL COMMUNICATIONS.

### ARTICLE I.

### *Progress in Dental Mechanism. No.* 4.

By Professor Austen.

In the first paper of this series, we briefly examined the Philosophy of Dental Progress ; in the second, assigned the three principal causes of its unexampled rapidity ; and in the third, gave illustrations of the last-named cause in connection with Dental Surgery ; or, as it is commonly termed Operative Dentistry. In the remaining papers, dental progress will be illustrated by the numerous improvements in Dental Mechanism, which have so wonderfully developed the resources of this difficult and useful branch of Dental Art.

We would, by way of introduction, suggest for general adoption, a change in dental nomenclature, used by us for years past in our lectures. The term "operative dentist," has about as much significance as that of "practical plumber," suggestive of plumbers "in theory," and of dentists who either cannot or will not work.

As both departments of dentistry are largely mechanical, it is proper that the names of each should embody this peculiarity. The dentistry of "Prevention and Cure,"

dealing mainly with living organs, is more analogous to the work of the general surgeon : hence the term SURGERY, although etymologically of purely mechanical import, is peculiarly significant. The dentistry of "Replacement," deals also with natural organs : but most of the work is done in the laboratory, and is analogous to that of the Mechanical Arts : hence the term MECHANISM, significant of the "thing made," is chosen to point out this relation. DENTISTRY embraces under this nomenclature, DENTAL SURGERY and DENTAL MECHANISM.

Our illustrations of progress, in the latter department, will be taken somewhat in the order of the operations as usually done. First in order, and paramount in importance, in the work of "replacement," is the *impression*, which is the starting point of the whole mechanism. Improvements in the material used and in the means of applying it, must, it need scarcely be said, increase the usefulness of dental art.

Impression cups consisted originally of the fingers and thumbs ; and nature's wax-holders are still the best, in certain cases, where a limited surface is to be copied, and where we require that delicacy of touch, to be found only in the ends of the fingers. Next in use were flat discs made of sheet tin (tinned-iron) with outer and inner rims soldered thereto, and the handle stiffened by an additional strip. To appreciate the superiority of the cups in present use (descriptions of which are unnecessary,) let us look for a moment at the properties essential to a good cup.

It must be of such shape as to retain the material. This rule applies to the use of plaster, which is not easily used in a badly shaped cup ; and which, when in thin layer, requires some provision for its adhesion to the cup in the act of removal. Applied to wax-holders, it implies sufficient depth of rim to give lateral support and condemns all upper cup with the palatine surface cut out. The old-fashioned cups were thus made ; but this shape suited the old form of plates, before the introduction of the broad "atmospheric" base. The re-introduction of this shape, or of cups with a large palatine opening, is due to a mistaken idea that secondary pressure, with the thumb or fingers upon the wax projecting through such space, is better than the uniform pressure of the solid cup. Steady uninterrupted pressure in one direction is essential to a good impression ; and the best cups are those which, permitting this, as far as possible, render unnecessary secondary pressure in any other direction.

Uniform thickness of the impression material is one of the most important of all the conditions of a good impression. Hence those cups are best which will give the most uniform space between cup and gum. The old cups were, in this respect, very defective ; but modern skill has given us almost all that can be asked. For impressions of full cases, upper or lower, four or five sizes to each jaw will meet the requirements of all ordinary cases. With such cups the depots are abundantly supplied : why do they not keep a similar supply of a form of cup equally indispensable? We refer to the square-edged cup for partial cases, shaped thus to receive the remaining teeth. If compelled exclusively to use any one form, we should select this: for it is much more difficult to take a partial impression in a rounded cup than to take a full impression in a square edged cup. An admirable form is in the market—our complaint is of the limited supply, due to the possible fact that the profession generally are not aware of the advantages of this form of cup.

Rigidity, sufficient to withstand the pressure in taking the impression, is the third and last requisite. In this also the old cups were very deficient ; many modern ones are unfit for any material except plaster, which requires no pressure. But manufacturers err sometimes on the other extreme and make cups too rigid. To make a few sizes most useful, we should be able to cut and bend to suit special cases, the dentist is here to blame often when the manufacturer has done his duty. When strong enough to stand the pressure, and soft enough to be bent; yet the dentist fails to take a good impression, because the shape of the cup cannot or will not fit to the mouth, as might be done in a few moments.

It is unnecessary to argue the superiority of wax impressions in cups well adapted to the mouth. A simple experiment will show that a thick mass of wax can not copy a surface as accurately as when the layer is thin. Moreover the pressure made by the operator is transmitted unequally; those parts of the gum lying over the thicker portions of wax will be less compressed than others. There may be cases in which we desire this inequality: but as a rule the compression must be uniform. Accuracy in the impression is too important to permit such uncertainties, as arise from a badly adapted wax-holder.

The *Gutta Percha Cup* for plaster impressions is another valuable improvement. This will be at once seen from the fact that there is no mouth, however irregular, or whatever the position of any remaining teeth, which may not be accurately taken in plaster by the use of gutta percha cups. Necessity compelled us ten years ago, in applying the vulcanite to "partial cases," to contrive and gradually improve this form of cup. Others may have felt, and in a similar manner overcome, the difficulty. If so they will be prepared to confirm our statements—that plaster impressions are essential to accuracy, in a large class of partial pieces, made of vulcanite, or other plastic material—and that, except in a few cases, gutta-percha cups are necessary, in taking these impressions.

Gutta-percha is also very useful for taking plaster impressions, in those exceptional cases where the brittania mouth cups cannot, without troublesome alteration, be used. Such cases were formerly (and are still by many careful operators) met by the swaging of special impression cups.—a most accurate method, but also a troublesome one. This brings us to one of the most important services rendered by inventive skill, the saving to the dentist of that which he cannot replace when lost—his time.

Some hesitate to purchase a dozen cups, and think twice before cutting and bending a cup for a given case, least it should be unfit for further use: they thus value the time spent in swaging a cup at less than fifty cents. There is no folly of early professional life, that the dentist will afterwards regret more deeply, than that of making for himself, what he can purchase already made, and usually much better made. Economy is the frequent excuse: whereas it is a most spendthrift extravagance. "Practice" is another plea: but this is not admissible, until it can be shown that he is skilled in the construction of all kinds of intricate dental mechanisms. "Nothing else to do" is a far worse apology. The *science* of dentistry claims every moment that can be spared from the practical details of the *art*.

Dental depots elevate dentistry, most of all in this, that they relieve the workers in the profession of much of that drudgery of mechanism which, in years past, consumed valuable time, yet was necessary to a given end. True, many will use this time simply for the extension of business and so reckon the benefit at a monied valuation. But others, satisfied with their usual gains, devote the spare hours to intellectual culture; to these the benefit is priceless.

We close our remarks upon impression cups, by noticing one of those so-called improvements, such as cumber our laboratories with a host of contrivances, as useless as complicated. The "double cups," in which the wax is cooled by injecting a stream of cold water between the two layers, are worse than useless. How any one, who has once cooled a wax impression with a small napkin, chilled in ice water, or enclosing a thin tablet of ice, can give up so simple and easy an operation, for the troublesome and cumbrous cups above named, passes our comprehension.

To sum up briefly the contributions to dental progress, which the inventive genius of the last twenty years has rendered, in the single matter of impression cups—1. It has greatly increased the number of good impressions, by putting within the reach of every one cups well adapted to this purpose. 2. It has for the skillful few, who knew how to make cups for themselves, saved hours of unimproving handwork, which they have been able to devoted to the cause of science. 3. It has made the taking of plaster impressions, possible in any case, and easy in many, where they were before thought impracticable. In our next paper, upon *impression materials*, this third point will be more fully brought out.

---

